# Oleuropein alleviates myocardial ischemia–reperfusion injury by suppressing oxidative stress and excessive autophagy via TLR4/MAPK signaling pathway

**DOI:** 10.1186/s13020-024-00925-x

**Published:** 2024-04-08

**Authors:** Jia He, Liting Huang, Kaili Sun, Jilang Li, Shan Han, Xiang Gao, Qin-Qin Wang, Shilin Yang, Wen Sun, Hongwei Gao

**Affiliations:** 1 College of Pharmacy, Jiangxi University of Chinese Medicine, Nanchang, 330004 China; 2 College of Pharmacy, Guangxi University of Chinese Medicine, Nanning, 530000 China; 3 Research Center for Traditional Chinese Medicine Resources and Ethnic Medicine, Jiangxi University of Chinese Medicine, Nanchang, 330004 China; 4 Guangxi Engineering Technology Research Center of Advantage Chinese Patent Drug and Ethnic Drug Development, Nanning, 530020 China; 5 State Key Laboratory of Stem Cell and Reproductive Biology, Institute of Zoology, Chinese Academy of Sciences, Beijing, 100101 China; 6 Institute for Stem Cell and Regeneration, Chinese Academy of Sciences, Beijing, 100101 China; 7 Beijing Institute for Stem Cell and Regenerative Medicine, Beijing, 100101 China

**Keywords:** Oleuropein, Myocardial ischemia–reperfusion, Oxidative stress, Autophagy, TLR4, MAPK

## Abstract

**Background:**

Myocardial ischemia/reperfusion injury (MIRI) is an important complication of reperfusion therapy, and has a lack of effective prevention and treatment methods. Oleuropein (OP) is a natural strong antioxidant with many protective effects on cardiovascular diseases, but its protective effect on MIRI has not yet been studied in depth.

**Methods:**

Tert-Butyl hydroperoxide (tBHP) was used to establish an in vitro oxidative stress model. Cell viability was detected by 3-(4,5)-dimethylthiahiazo (-z-y1)-3,5-di-phenytetrazoliumromide (MTT) and lactate dehydrogenase (LDH). Flow cytometry and fluorescence assays were performed for evaluating the ROS levels and mitochondrial membrane potential (MMP). Immunofluorescence analysis detected the NRF2 nuclear translocation and autophagy indicators. Further, Western blotting and quantitative real-time PCR were performed to evaluate the expression levels of proteins and mRNAs. Molecular docking, CETSA, and molecular interaction analysis explored the binding between OP and TLR4. The protective effects of OP in vivo were determined using a preclinical MIRI rat model.

**Results:**

OP protected against tBHP-treated injury, reduced ROS levels and reversed the damaged MMP. Mechanistically, OP activated NRF2-related antioxidant pathways, inhibited autophagy and attenuated the TLR4/MAPK signaling pathway in tBHP-treated H9C2 cells with a high binding affinity to TLR4 (*K*_*D*_ = 37.5 µM). The TLR4 inhibitor TAK242 showed a similar effect as OP. In vivo, OP could alleviate cardiac ischemia/reperfusion injury and it ameliorated adverse cardiac remodeling. Consistent with in vitro studies, OP inhibited TLR4/MAPK and autophagy pathway and activated NRF2-dependent antioxidant pathways in vivo.

**Conclusion:**

This study shows that OP binds to TLR4 to regulate oxidative stress and autophagy for protecting damaged cardiomyocytes, supporting that OP can be a potential therapeutic agent for MIRI.

## Introduction

Ischemic heart disease (IHD) has always been a major disease threatening human health with a high morbidity and mortality rate and has surpassed cancer as a leading cause of death [1]. Treatment methods such as arterial bypass grafting, thrombolysis, and percutaneous coronary intervention can restore myocardial blood supply; however, subsequent ischemia–reperfusion may lead to further damage to the myocardium. Thus, myocardial ischemia/reperfusion injury (MIRI) seriously affects patient recovery and prognosis [2]. Currently, research shows that a number of natural products or traditional Chinese medicine compounds have the potential to treat MIRI, which may open up new avenues for MIRI treatment [3, 4].

Oxidative stress induced by steeply elevated ROS levels during the reperfusion phase is considered to be the main mechanism underlying MIRI [5]. Oxidative stress can cause cardiomyocyte injury and apoptosis, participate in ventricular remodeling, promote the formation and development of cardiomyopathies, and cause cardiac dysfunction. The hyperfunctioning cardiomyocytes are more sensitive to oxidative stress damage, forming a vicious circle that further aggravates the injury [6]. In addition, excessive ROS during the reperfusion phase can also overactivate autophagy. Autophagy is a widespread normal physiological process, but excessive autophagy can phagocytize and degrade normal proteins and organelles, damage the normal physiological conditions of cells, and subsequently result in autophagic cell death which is often accompanied by apoptosis and necrosis [7]. Therefore, inhibition of oxidative stress and autophagy during the ischemia–reperfusion phase may play a protective role in cardiomyocytes.

Mitogen-activated protein kinase (MAPK) is a family of serine/threonine kinases that can mediate intracellular signals in response to various stimuli. Studies have shown that the MAPK signaling pathway is involved in MIRI pathogenesis through the activation of three key subfamilies, c-Jun N-terminal kinase (JNK), extracellular regulated protein kinase (ERK1/2), and P38 molecules [8]. Activation of MAPKs supports ROS generation and subsequent oxidative stress and autophagy [9, 10]. TLR4, which is one of the representative receptors of the innate immune response, is the first discovered member of the toll-like receptors (TLRs) family. Further, TLR4 widely exists on the myocardial cell membrane and participates in various physiological and pathological processes. Studies have indicated that TLR4 plays a central role in ischemia/reperfusion injury. As an upstream factor of MAPK, TLR4 activates the MAPK signaling pathway by recognizing various endogenous ligands and some components of the extracellular matrix to regulate the transcription of downstream related genes [11]. Therefore, the TLR4/MAPK signaling pathway may be a potential therapeutic strategy for MIRI.

Oleuropein (OP) is the main active ingredient isolated from *Ilex pubescent var. kwangsiensis* Hand. –Mazz [12]. It is an iridoid phenolic compound with various pharmacological effects such as anti-tumor [13], anti-inflammatory [14], anti-oxidative [15], detoxifying [16], blood sugar lowering [17], blood pressure lowering [18], blood fat lowering [19], and organ protective [20] effects. Among them, the protective effect of OP on cardiovascular diseases is particularly prominent, but its protective effect and mechanism on MIRI have not yet been fully elucidated. This study aims to investigate the protective effect of OP on MIRI and whether it regulates oxidative stress and autophagy as the underlying mechanism.

## Materials and methods

### Reagents

Oleuropein was obtained from Chengdu Push Bio-tech Co., Ltd (32619-42-4, Chendu, China). Dulbecco's Modified Eagle Medium (DMEM), fetal bovine serum (FBS), penicillin/treptomycin (P/S), and 0.25% Trypsin–EDTA (1 ×) were obtained from Gibco (California, USA). Tert-Butyl hydroperoxide (tBHP) and 3-[4, 5]-dimethylthiahiazo (-z-y1)-3,5-di-phenytetrazoliumromide (MTT) were purchased from Sigma-Aldrich (298-93-1, Taufkirchen, Germany). Chloroquine (CQ) and TAK242 were purchased from MedChemExpress (Shanghai, China). Dimethyl sulfoxide (DMSO) was purchased from Solarbio (D8370, Beijing, China). Trizol Total RNA Extraction Regent (G3013), SweScript RT I First Strand cDNA Synthesis Kit (G3330), and 2 × Fast SYBR Green qPCR MasterMix (G3324) were purchased from Servicebio (Wuhan, China). Further, trichloromethane was purchased from Sinopharm Chemical Regent Co., Ltd. (Shanghai, China). LDH Cytotoxicity Assay Kit was purchased from Beyotime (C0016, Shanghai, China). Finally, 2′,7′-Dichlorodihydrofluorescein diacetate (DCFH2-DA) and JC-1 fluorescent probes were obtained from MedChemExpress (Shanghai, China), and Stub-RFPSens-GFP-LC3 was purchased from Genechem (Shanghai, China).

NRF2 (ab31163) and NQO1 (ab80588) antibodies were purchased from Abcam (Cambridge, UK). Keap1 (4678s), HO-1(70081s), GAPDH (5174s), PARP (9832s), LC3B (2775s), P62(39749s), p-P38(4511s), P38 (9212 s), p-ERK1/2 (4370s), p-JNK (9255s), and JNK (9252s) antibodies and the secondary antibodies including horseradish peroxidase (HRP)-conjugated goat anti-rabbit IgG (7074) were purchased from Cell Signaling Technology (Danvers, MA, USA). TLR4(48–2300) antibody was purchased from Invitrogen (California, USA). ERK1/2 (AF6248) antibody and goat anti-mouse IgG Fluor® 488 Conjugate (S0017) were purchased from AFFINITY (Jiangsu, China). Pierce™ BCA Protein Assay Kit (23225) was purchased from ThermoFisher (California, USA). QuantiCyto® Rat IL-6 ELISA kit (ERC003) and QuantiCyto® Rat TNF-α ELISA kit (ERC102a) were purchased from NEOBIOSCIENCE (Shenzhen, China).

### Cell culture

H9C2 and HEK293T cells were purchased from the Cell Bank of the Chinese Academy of Sciences (Shanghai, China), and were cultured in DMEM complemented with 10% FBS and 1% P/S. The abovementioned cells were maintained in a 37 °C incubator filled with 5% CO_2_.

### Cell viability assay

H9C2 cells were seeded into a 96-well plate at the density of 6000 cells per well overnight. The OP solution used in cells was first dissolved in DMSO to prepare a 40 mM stock solution, and then diluted with the cell culture medium to the required concentration. After that, OP was added to H9C2 cells at doses from 10 to 80 μM and incubated for 24 h. After that, 100 μL of 1 mg/mL MTT was added followed by incubation for another 4 h at 37 °C. The MTT in each well was replaced with DMSO for 15 min at room temperature and the plate was then evaluated using a microplate reader (SYNERGYH1, Bio Tek, USA) by measuring absorbance of 570 nm.

### LDH release assay

H9C2 cells were seeded into a 96-well plate at the density of 6000 cells per well overnight. This was followed by a pretreatment with OP at different concentrations for 2 h followed by treatment with tBHP for another 4 h. The supernatants were collected to detect the LDH release level according to the manufacture’s protocol. The absorbance was measured at 450 nm using a multimode plate reader.

### Flow cytometry

H9C2 cells were seeded into a 12-well plate at the density of 1 × 10^5^ cells per well overnight. This was followed by a pretreatment with OP at different concentrations (10, 20, and 40 μM) for 2 h followed by treatment with tBHP for another 2 h. The cells were labeled with DCFH2-DA probe (1 μM) for 30 min and then washed and collected for detection by the FACSMelodyTM Cell Sorter (BD bioscience, USA).

### Fluorescence assay

H9C2 cells were seeded into a 96-well plate at the density of 6000 cells per well overnight, followed by pretreatment with OP at the concentration of 40 μM for 2 h followed by treatment with tBHP for another 2 h. The ROS level was measured using DCFH2-DA (1 µM), and the MMP was measured using JC-1 solution (10 µM), following the manufacturer’s instructions. The fluorescence was observed under a fluorescence microscope (Leica, Wetzlar, Germany).

### Immunofluorescence analysis

H9C2 cells were seeded into a confocal dish (SPL, Pocheon, Korea) at the density of 1.2 × 10^5^ cells per well overnight, followed by pretreatment with OP at the concentration of 40 μM for 2 h and treatment with tBHP for another 2 h. The immunofluorescence levels of Nrf2, LC3B, and P62 were measured using an anti-Nrf2 antibody, anti-LC3B antibody, and anti-P62 antibody overnight at 4 °C, followed by incubation with goat anti-rabbit Alexa Fluor 488 secondary antibody (1:200) for 2 h at room temperature. Subsequently, the cells were additionally stained for 5 min with Hoechst 33,342 (1 μM) before being imaged. The cells were imaged using a confocal laser microscope (Leica, Wetzlar, Germany).

### Autophagic flux assay

According to the manufacturer’s protocol, H9C2 cells were infected with lentiviruses for 36 h and the expression of GFP and RFP was observed under a fluorescence microscope (Leica). Afterward, the cells were fixed with 4% PFA and stained with Hochest33342 for 5 min at room temperature away from light. Finally, the cells were imaged using a confocal laser microscope.

### Western blot assay

The cells were lysed by RIPA lysis buffer with 1% cocktail and 1% phenylmethylsulfonyl fluoride (PMSF) and separated by centrifugation. Total proteins were collected, and the concentration of proteins was measured by the BCA protein assay kit. The quantified proteins were separated by SDS-PAGE and then transferred onto a PVDF membrane. After blocking with 5% non-fat dry milk solution for 1 h at room temperature, the membranes were incubated with the primary antibody (1:1000 dilutions) at 4 °C over 16 h. Then, the blot was treated with HRP-conjugated secondary antibodies (1:5000 dilutions) and visualized using the ChemiDoc XRS + System (California, USA). The gray-scale values of related proteins were quantified by Image J. The cytoplasmic and nuclear proteins were extracted using a nuclear and cytoplasmic protein extraction kit (Beyond time, Shanghai, China) according to the manufacturer’s instructions and the protein level detection was as described above.

### Quantitative real-time PCR (qRT-PCR) assay

H9C2 cells were seeded into a dish (SPL, Pocheon, Korea) at the density of 1.2 × 10^5^ cells per well overnight, followed by pretreatment with OP at the concentration of 40 μM for 2 h and treatment with tBHP for another 2 h. Total RNA was extracted and 1 μg RNA was used for qRT-PCR analysis. Nrf2, HO-1, NQO1, and GAPDH oligonucleotide primers were as follows (Table 1)

**Table 1:** Primer Sequence (5′-3′)

Gene name	Base sequence
*Nrf2*	F: CAGTCAGCGACGGAAAGAGTA
	R: TGTGGGCAACCTGGGAGTAG
*HO1*	F: CTCCGATGGGTCCTTACACTC
	R: ATAGGCTCCTTCCTCCTTTCC
*NQO1*	F: GCTGGTTTGAGCGAGTGTTC
	R: TGGAGTGTGCCCAATGCTATAT
*GAPDH*	F: CAGGAGGCATTGCTGATGAT
	R: GAAGGCTGGGGCTCATTT

### Molecular docking

The 3D structure of OP was downloaded through Pubchem. The PDB structure of TLR4 (PDB number: 3FXI) was downloaded through PDB, and the structure was processed using Autodock software (https://autodocksuite.scripps.edu/adt/) for hydrogenation and dehydration and finally molecular docking.

### Cellular thermal shift assay (CETSA)

The biochemical mechanism of HEK293T cells is suitable for expressing most mammalian proteins requiring post-translational modifications and protein folding, as their protein structure closely resembles that of the human body [21]. Therefore, HEK293T cells are often chosen in experiments such as CETSA to detect the binding of drugs to proteins in their native conformations [22]. Total proteins were obtained from HEK293T cell lysate lysed with RIPA lysis buffer (1% PMSF and 1% cocktail). Then, the respective cell lysates were co-incubated with vehicle control (DMSO) or OP (40 μM) for 0.5 h on ice and then centrifuged at 12,000 rpm for 20 min at 4 °C. Following this, the protein concentration was quantified using a BCA kit. The supernatant was divided into six parts on average and heated at different temperatures (40, 44, 48, 52, 56, and 60 ℃) for 3 min followed by cooling for 30 s at room temperature, and detection by Western blotting.

### Molecular interaction analysis

Biacore X100 (Cytiva, United States) was used for measuring the interaction of OP with TLR4. HBS-EP (Cytiva, United States) was used as the working buffer to dilute the TLR4 recombinant protein (Sino Biological, China) to a final concentration of 20 μg/mL. Next, the CM5 chip was activated and TLR4 recombinant protein was coupled with the CM5 chip by the amino coupling method. Furthermore, OP was diluted with the HBS-EP buffer to 100, 50, 25, 12.5, 6.25, 3.125, and 1.5625 μM. Kinetic experiments were performed using the kinetic and affinity methods in the template of the Kinetic Analysis Wizard to analyze interactions between the ligand and the receptor. The obtained data were fitted according to the analysis software, with time as the abscissa and the response value as the ordinate to calculate the binding kinetics between OP and TLR4.

### Myocardial ischemia–reperfusion animal model

Animal experiments were approved by the Ethics Committee on Laboratory Animal Management of Guangxi University of Chinese Medicine (Approval Document No. SYXK Gui-2019-0001). Healthy male Sprague–Dawley rats (SPF grade, 6–8 weeks old, body weight 220–250 g) were purchased from Hunan SJA Laboratory Animal Co., Ltd (Hunan, China, animal license number: SCXK Xiang-2021-0002) and were housed under specific pathogen–free (SPF) conditions at 23–25 °C with 40–50% humidity and free access to food and water. After three days of adaptive feeding, the rats were randomly divided into five groups (n = 10 per group): the sham group, the I/R group, the OP group (OP-L:10 mg/kg and OP-H:40 mg/kg) and the Verapamil group (20 mg/kg) [23]. OP solution and verapamil solution used in rats were formulated with normal saline at 1 mg/mL, 4 mg/mL, or 2 mg/mL, and OP was then administered to mice via an intraperitoneal injection (*i.p.*). Verapamil was administered to mice by intragastric administration (*i.g.*); both solutions were administered once daily for seven consecutive days. The sham and model groups were administered the same amount of normal saline. The needle was inserted 2 mm from the lower border of the left atrial appendage, and the left anterior descending coronary artery was ligated. At this time, it was observed that the color of the rat's heart surface turned purple. After 30 min, the ligation was removed, and the heart blood was reperfused for 2 h. During this period, the color of the heart's surface returned to red. In the sham group, needles were only inserted without ligation. After reperfusion, the rats were sacrificed, and tissues were collected for subsequent experiments.

### Blood routine

After the rats were sacrificed, blood was collected from the abdominal aorta for routine blood testing using the Auto Hematology Analyzer (BC-500, Mindray, Shenzhen, China).

### Enzyme-linked immunosorbent assay (ELISA)

Rat blood was collected and left at room temperature for 2 h, centrifuged at 3000 rpm for 15 min at low temperature to get the upper serum, and the levels of IL-6 and TNF-α in the serum were detected using ELISA kits according to the kit and instrument instructions.

### 2,3,5-triphenyltetrazolium chloride (TTC)-evens’ blue double staining

After 2 h of reperfusion, rats were anesthetized again, and the coronary artery was ligated again at the position of ischemia; 0.3 mL of 1% Evans blue was injected from the apex of the heart to turn the non-ischemic myocardium blue, and the rest of the myocardium was regarded as ischemia danger zone. The heart was then immediately resected; the residual blood was washed with pre-cooled PBS buffer, blotted dry with filter paper, and placed in a freezer at − 20 °C for 30 min. Subsequently, 5–6 slices were evenly cut across the direction perpendicular to the long axis of the left ventricle, followed by soaking sequentially in warm 1% TTC solution, and incubation at 37 °C in dark for 10 min. At this time, the infarct area was grayish-white.

### Hematoxylin and eosin (HE) staining

Other parts of rat hearts were fixed with 4% paraformaldehyde for 24 h at room temperature and prepared for paraffin sectioning. The fixed tissues were routinely dehydrated and transparent, embedded in paraffin and sliced (5 μm), dried, dewaxed, hydrated, and stained with hematoxylin for 10 min. The tissues were then washed with distilled water, differentiated with 1% hydrochloric acid ethanol for 10 s, washed with distilled water again, and stained with 1% eosin for 3 min. This was followed by washing, and routine dehydration and mounting. The pathological changes in the myocardial tissue were finally observed under a microscope.

### Data analysis

Each experiment was performed three times. Student’s unpaired t-test and one-way ANOVA in GraphPad Prism were used for statistical analysis in GraphPad Prism 9 (GraphPad Software, San Diego, CA). *P* < 0.05 was considered statistically significant.

## Results

### OP improves cell viability and mitochondrial homeostasis in tBHP-induced H9C2 cells

**Fig. 1:**
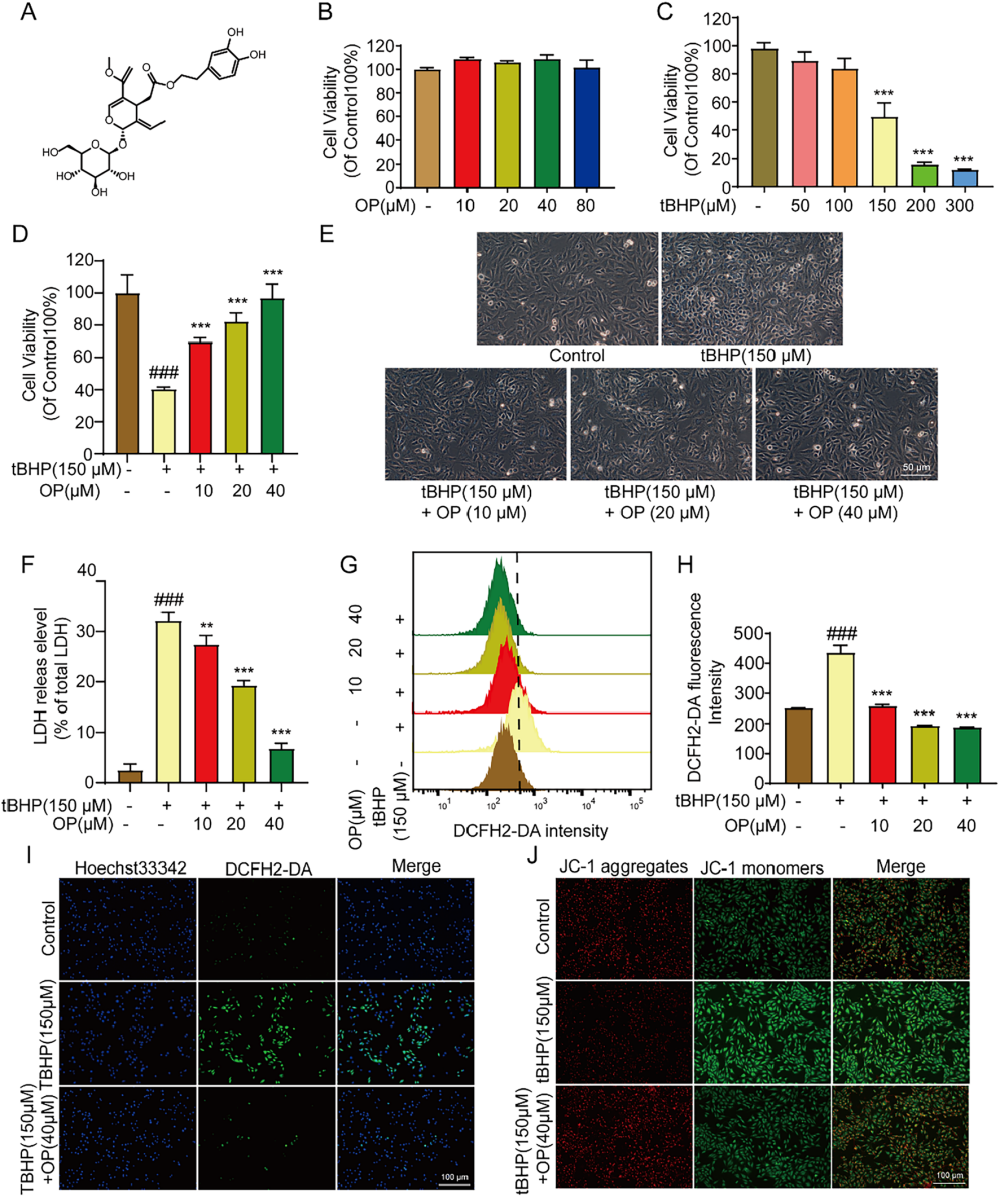
OP improves the cell viability and mitochondrial homeostasis in tBHP-induced H9C2 cells. **A** The chemical structure of OP. **B** H9C2 cells were treated with OP (5, 10, 20, 40, 80 μM) for 24 h, the cytotoxicity was detected by MTT assay. **C** H9C2 cells were treated with tBHP (50, 100, 150, 200, 300 μM) for 4 h, the cell viability was detected by MTT assay. **D** H9C2 cells were pretreated with OP (10, 20, 40 μM) for 2 h and then acted with tBHP (150 μM) for 4 h, the cell viability was detected by MTT assay. **E** The effect of OP in tBHP- induced changes of Cell morphology. **F** The LDH release level. **G** Flow cytometry detected the level of ROS. **H** Statistics of flow cytometry. **I** H9C2 cells were stained with DCFH2-DA to detect the ROS level. **J** H9C2 cells were stained with JC-1 probe to detect the MMP level. All data are expressed as mean ± SEM, n = 3. ^###^*P* < 0.001 *vs* control group; ^*^P < 0.05, ^**^P < 0.01, ^***^P < 0.005 *vs* tBHP group

As shown in Fig. 1A, OP is a secoiridoid glycoside polyphenolic compound. Our results showed that the concentrations of OP from 10 to 80 μM showed no significant toxicity on H9C2 cells (Fig. 1B), whereas tBHP decreased the cell viability in a dose-dependent manner; the cell death reached approximately 50% at a dose of 150 μM (Fig. 1C). Therefore, 150 μM tBHP was the concentration chosen for the following experiments. OP (10, 20, and 40 μM) could protect H9C2 cell viability, restore cell morphological changes and reduce the LDH release level in tBHP-damaged H9C2 cells (Fig. 1D–F). The ROS level reflects the degree of oxidative stress damage. Figure 1G–I shows that tBHP stimulated a sharp increase in ROS levels and OP significantly reduced the levels of ROS. In addition, tBHP caused the loss of mitochondrial membrane potential (MMP), which was distinctly reversed with OP treatment. Taken together, OP suppressed oxidative stress-induced cardiomyocyte damage by inhibiting ROS levels and restoring mitochondrial homeostasis.

### OP activates Nrf2 signal pathway in tBHP-induced H9C2 cells

**Fig. 2:**
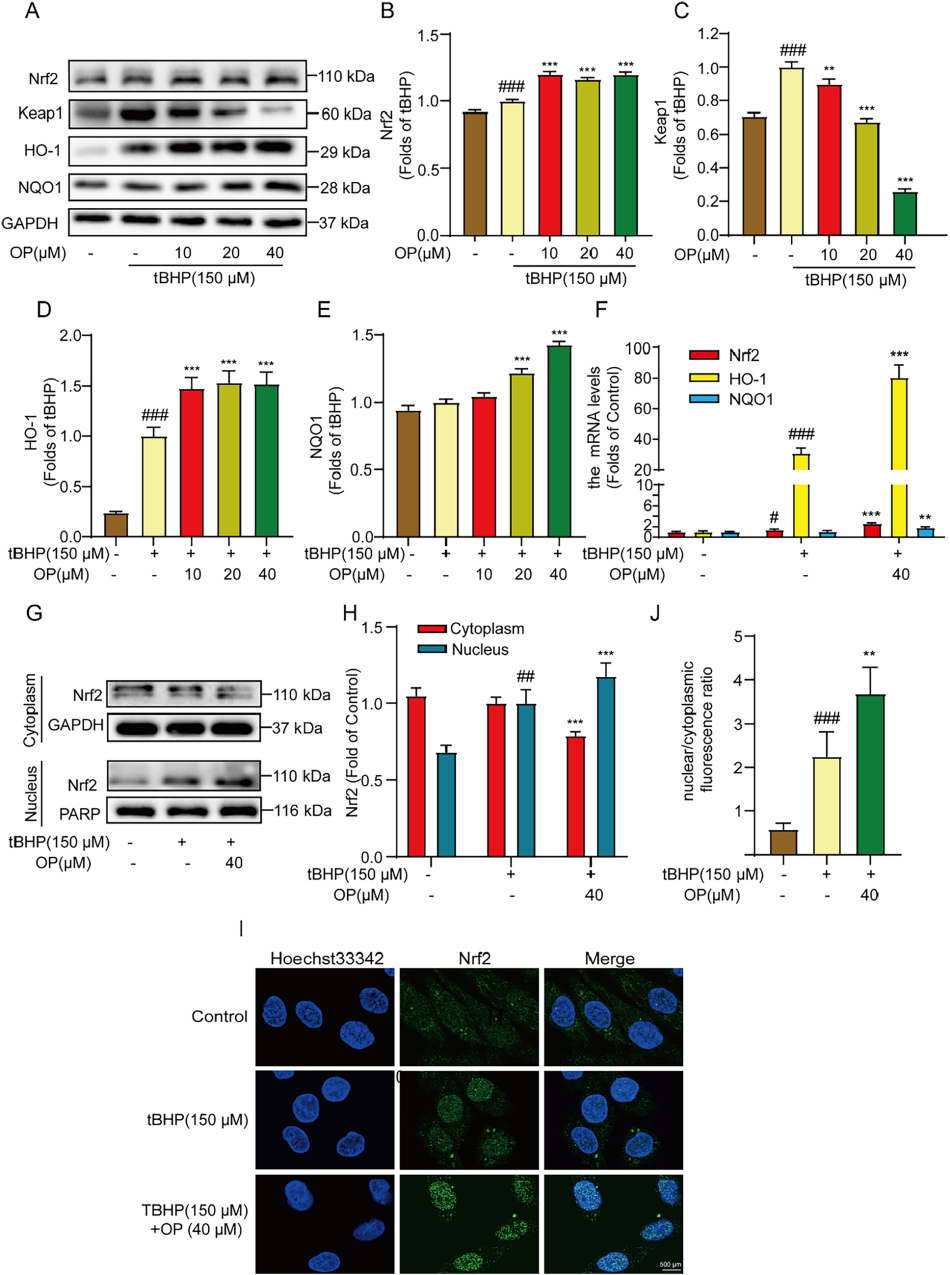
OP actives Nrf2 signal pathway in tBHP-induced H9C2 cells. **A** The effect of OP on the protein expressions of Nrf2, Keap1, HO-1, and NQO1 in tBHP-induced H9C2 cells as indicated. **B**–**E** The statistics of protein gray value. **F** Qrt-PCR detected the effect of OP on the mRNA levels of Nrf2, HO-1, and NQO1 in tBHP-induced H9C2 cells. **G** The Nrf2 protein levels in cytoplasm and nucleus were detected by western blot. **H** The statistics of Nrf2 protein level. **I** The level of Nrf2 nuclear translocation was detected by immunofluorescence. **J** Statistics of the ratio of nuclear and cytoplasmic fluorescence. All data are expressed as mean ± SEM, n = 3. ^#^*P* < 0.05, ^##^*P* < 0.01, ^###^*P* < 0.005 *vs* Control; ^**^P < 0.01, ^***^P < 0.005 *vs* tBHP

Nrf2 is an important transcription factor that regulates the oxidative stress response of cells by inducing and regulating the expression of a series of antioxidant proteins to maintain the body’s redox dynamic balance [24]. As shown in Fig. 2A–E, tBHP increased the protein levels of Nrf2, Keap1, HO-1, and NQO1; OP treatment then decreased the Keap1 level and further increased Nrf2, HO-1, and NQO1 levels. RTq-PCR showed the analogous result indicating that OP further elevated the mRNA levels of Nrf2, HO-1, and NQO1 based on increased tBHP induction. The regulation of downstream signaling by Nrf2 requires Nrf2 translocation into the nucleus. The results showed that OP increased the nuclear Nrf2 protein level while decreasing the cytoplasmic protein level, supporting Nrf2 translocation from the cytoplasm to the nucleus (Fig. 2H–J).

### OP inhibits autophagy in tBHP-induced H9C2 cells

**Fig. 3:**
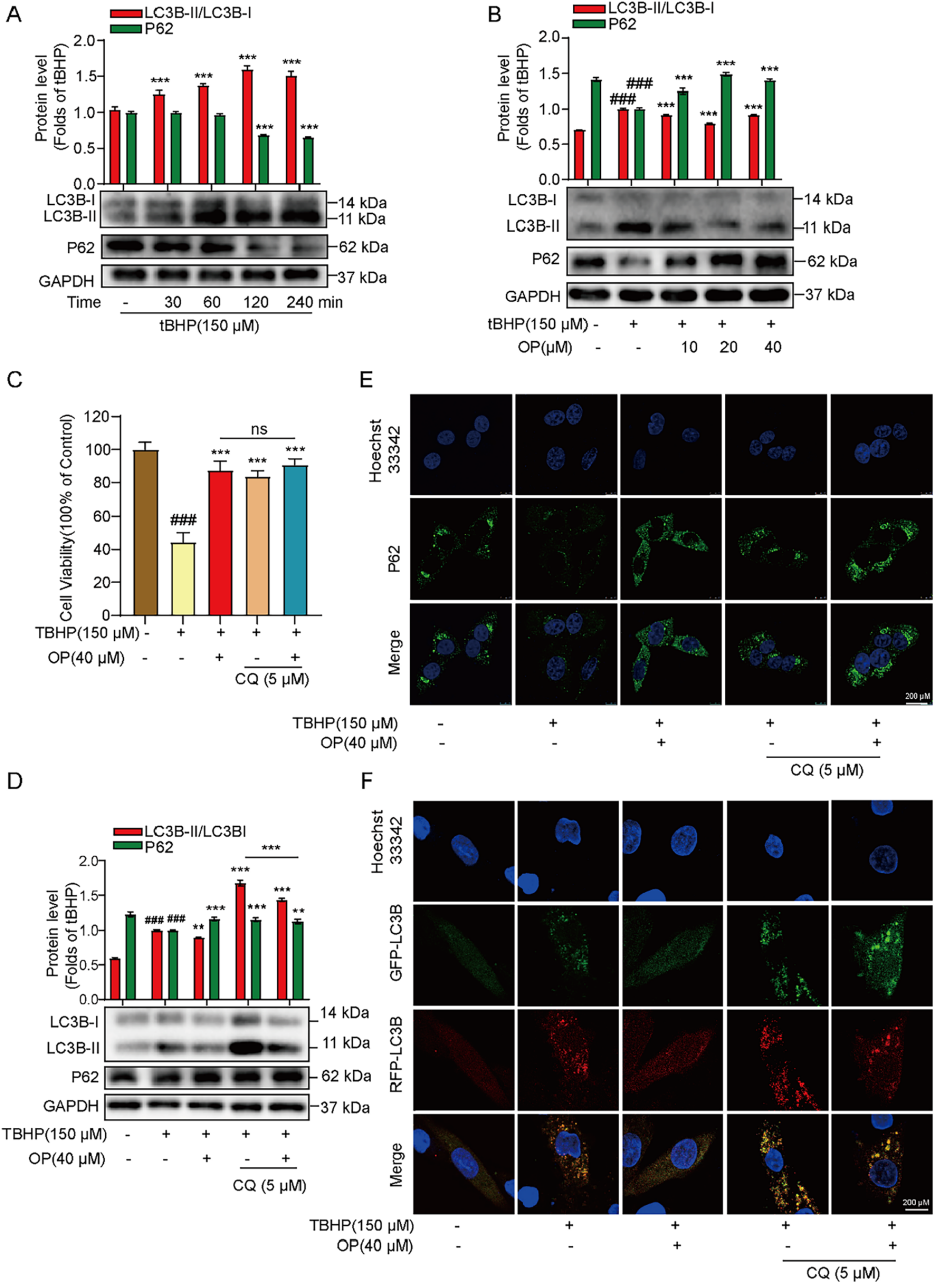
OP inhibits autophagy in tBHP-induced H9C2 cells. **A** The LC3B and P62 proteins expression in tBHP treatment H9C2 cells for different time points of 30, 60, 120, 240 min. **B** The effect of OP on the protein expressions of LC3B and P62 in tBHP-induced H9C2 cells as indicated. **C** H9c2 cells were pretreated with or without CQ (5 μM) for 2 h and then treated or untreated with OP for other 2 h, after which acted with tBHP (150 μM) for 4 h, the cell viability was detected by MTT assay. **D** The effect of OP or CQ on the protein expressions of LC3B and P62 in tBHP-induced H9C2 cells as indicated. **E** The level of P62 was detected by immunofluorescence. **F** The fluorescence levels of LC3B in H9C2 cells infected with stubRFP-sensGFP-LC3 lentivirus. All data are expressed as mean ± SEM, n = 3. ^###^*P* < 0.005 *vs* Control; ^**^P < 0.01, ^***^P < 0.005 *vs* tBHP

When the body suffers from ischemia/reperfusion injury, autophagy levels are significantly upregulated. Excessive autophagy aggravates myocardial injury at the reperfusion stage, resulting in autophagic cell death, which is often accompanied by apoptosis and cell necrosis [25]. ROS accumulation is a major promoter of autophagy during reperfusion. The explosive ROS generation after myocardial ischemia–reperfusion can promote the lipidation of LC3 and formation of autophagosomes, promoting the activation of autophagy. As shown in Fig. 3A, tBHP induced an increased ratio of LC3B-II/LC3B-I and decreased P62 (sequestosome 1, SQSTM1) protein level. Simultaneously, OP treatment significantly reversed the result, indicating that OP can regulate autophagy in tBHP-treated H9C2 cells (Fig. 3B).

CQ is an autophagy inhibitor that mainly inhibits autophagy by disrupting the fusion of autophagosomes and lysosomes [26]. These results showed that CQ can protect from tBHP-induced damage in H9C2 cells (Fig. 3C). Moreover, after CQ blocked autolysosome formation, LC3B-II and P62 were further accumulated in the cells, suggesting that tBHP promoted the formation of autophagosomes but didn’t inhibit their degradation to induce excessive autophagy. On the premise that CQ further increased LC3B-II, OP decreased the protein expression of LC3B-II, indicating that OP could inhibit autophagy at an early stage (Fig. 3D).

P62 fluorescence was inhibited by tBHP, but OP and CQ both reversed this inhibitory state, further corroborating the results reported previously (Fig. 3E). GFP is an acid-sensitive protein, whereas mRFP is a stable fluorescent expression group. When autophagosomes fuse with lysosomes to form autolysosomes, GFP is quenched because of the acidic environment inside lysosomes, indicating the smooth formation of autolysosomes. At the same time, mRFP is always stably expressed, so the process of autophagy flow is evaluated by the ratio of bright spots between GFP and mRFP. Fluorescence results (Fig. 3F) showed that under untreated conditions, red and green fluorescence were diffuse; tBHP induced cells to produce red/green co-localized punctate aggregations (yellow spots), indicating the occurrence of autophagy. Both yellow and red spots were significantly reduced after OP treatment, which means that OP inhibited the production of autophagosomes and the development of autophagy. CQ further promotes red/yellow spot formation, but is also suppressed by OP. The abovementioned results illustrated that the protective effect of OP may be carried out by inhibiting the excessive autophagy induced by tBHP in H9C2 cells.

### OP binds to TLR4 and regulates the MAPK pathway

**Fig. 4:**
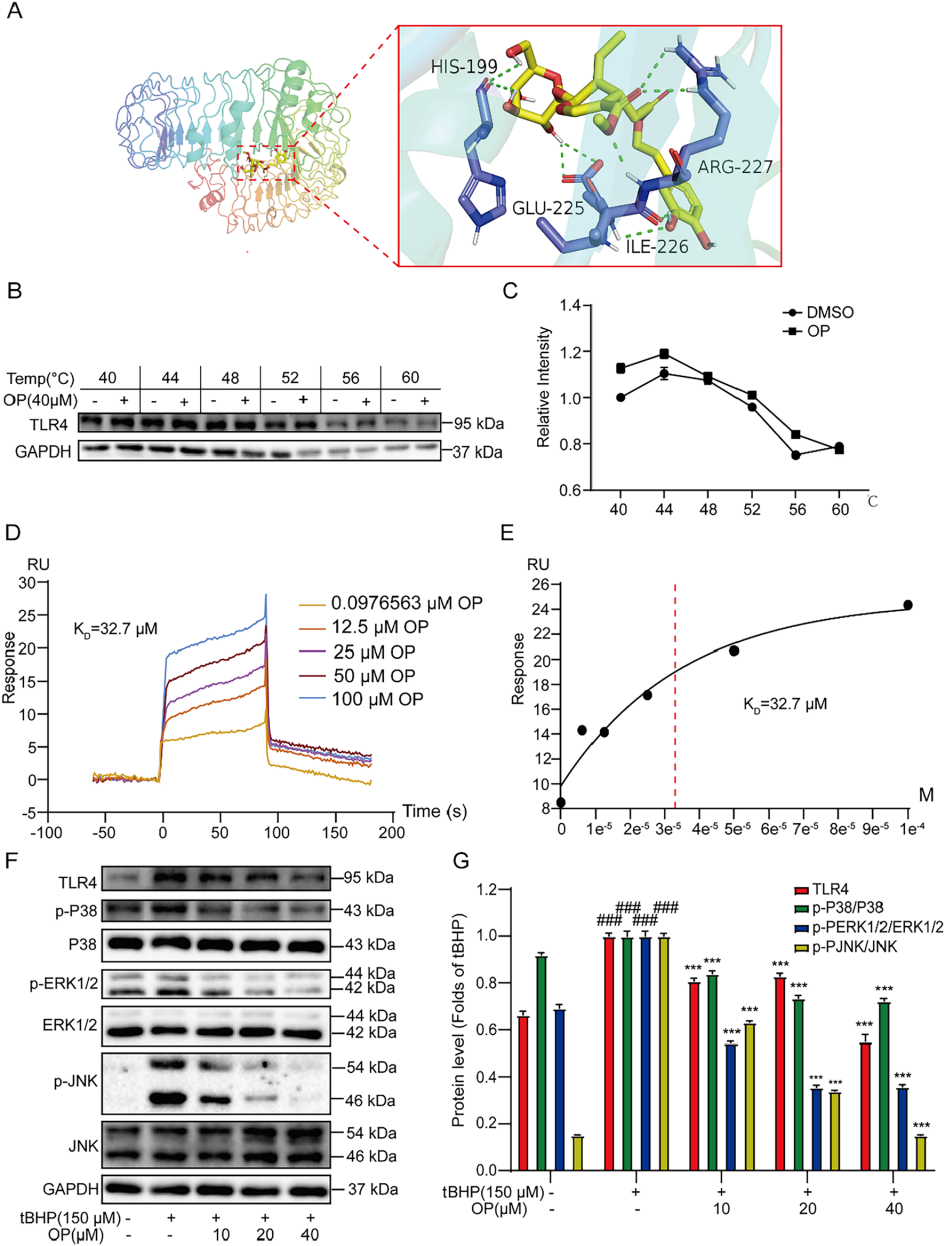
OP binds to TLR4 and regulates MAPK pathway. **A** Molecular docking data analysis of OP and TLR4 using autodock software. **B** The CETSA experiment further confirmed the interaction of OP and TLR4 (n = 3). **C** The statistics of protein levels of (B). **D**, **E** Biacore X100 detected the kinetics/Affinity of OP with TLR4. **F** The effect of OP on the level of TLR4 and the phosphorylation levels of P38, ERK1/2 and JNK in tBHP-induced H9C2 cells (n = 3). **G** The statistics of protein levels of (F). ^###^*P* < 0.005 *vs* Control; ^***^P < 0.005 *vs* tBHP (or DMSO)

TLR4 widely exists on the myocardial cell membrane and participates in various physiological and pathological processes as one of the representative receptors of the innate immune response [27]. During ischemia/reperfusion injury, TLR4 acts as an upstream factor to activate MAPK signaling pathway. Studies have shown that TLR4-associated MAPK signaling pathway is involved in autophagy and oxidative stress [28]. Furthermore, ROS can act as a second messenger to activate the MAPK signaling pathway and also be affected by the activation [29]. Therefore, we further examined whether TLR4/MAPK was involved in the effect of OP. The molecular docking data of the effect of OP on TLR4 was analyzed by the Autodock software. The result showed that OP interacts with TLR4 through the amino acids HIS199, GLU2525, ILE226, and ARG227. The binding energy reached − 7.0 kcal/mol, indicating that OP has strong docking activity with TLR4 (Fig. 4A). Furthermore, the CETSA-WB experiment was performed to further confirm the interaction between OP and TLR4 (Fig. 4B, C). Similarly, Biacore X100 SRP assay detected the kinetics/affinity of OP and TLR4. The results showed that the *K*_*D*_ value was 32.7 μM, which further indicated strong interaction of OP with TLR4 (Fig. 4D, E). Moreover, OP inhibited the activated TLR4/MAPK signaling pathway in tBHP-induced H9C2 cells. These data indicate that OP targets TLR4 and inhibits the MAPK signaling pathway in tBHP-stimulated H9C2 cells.

### TLR4 inhibitor TAK242 reverses the tBHP-induced cellular damage and regulates TLR4/MAPK/Nrf2 pathway and autophagy in H9C2 cells

**Fig. 5:**
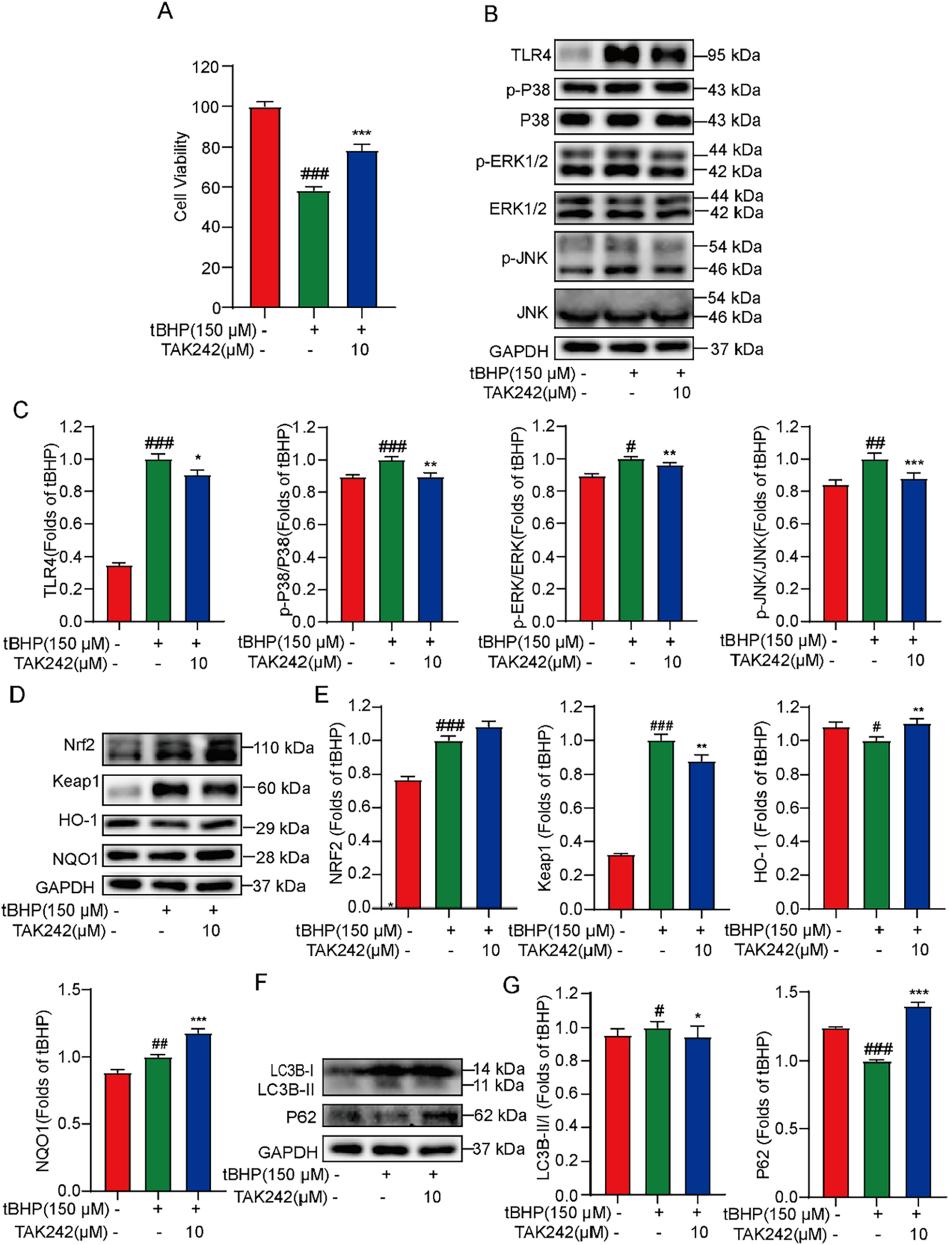
TAK242 suppresses the TLR4/MAPK/Nrf2 pathway and autophagy in tBHP-induced H9C2 cells. **A** H9C2 cells were pretreated with TAK242 (10 μM) for 1 h and then acted with tBHP (150 μM) for 4 h, the cell viability was detected by MTT assay. **B** The effect of TAK242 on the level of TLR4, p-P38, p-ERK1/2, p-JNK. **C** The statistics of protein levels of (B). **D** The effect of TAK242 on the level of Nrf2, Keap1, HO-1, NQO1. **E** The statistics of protein levels of (D). **F** The effect of TAK242 on the level of LC3B, P62. **G** The statistics of protein levels of (F). ^#^*P* < 0.05, ^##^*P* < 0.01, ^###^*P* < 0.005 *vs* Control; ^*^P < 0.05, ^**^P < 0.01, ^***^P < 0.005 *vs* tBHP (or DMSO)

To further confirm the contribution of TLR4 in oxidative stress and autophagy signaling pathways in tBHP-induced H9C2 cells, TAK242, a TLR4 inhibitor, was used for inhibiting TLR4 function [30]. As shown in Fig. 5A, TAK242 significantly reversed the decreased cell viability significantly. Moreover, TAK242 also inhibited the tBHP-activated phosphorylation of MAPKs (Fig. 5B, C), promoted the Nrf2/HO-1/NQO1 pathway (Fig. 5D, E), and decreased autophagy in tBHP-induced H9C2 cells (Fig. 5F, G). These results suggested that TLR4 indeed served a central role in the regulation of oxidative stress and autophagy in tBHP-induced H9C2 cells.

### OP alleviates MIRI in rats

**Fig. 6:**
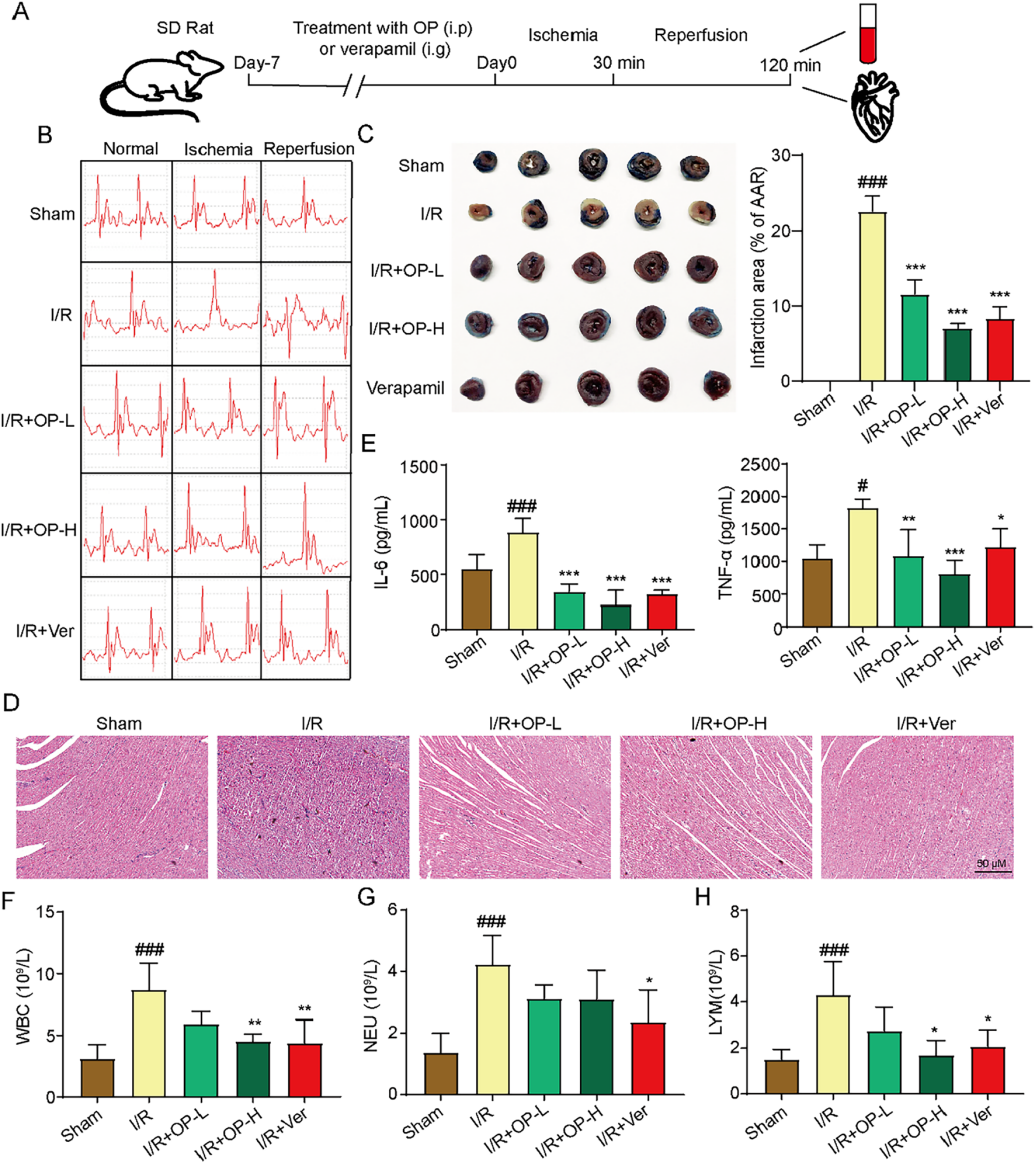
OP alleviates myocardial ischemia reperfusion injury. **A** The Animal Experiment Schedule. **B** The electrocardiogram of rats (n = 10). **C** The TTC-Even's blue double staining of heart tissue. Infarct size (%) was expressed as the percentage of infarct area relative to the area at risk (AAR). the nonischemic section shown in blue area, red represent risk area, and the infarct region is stained white (n = 3). **D** The HE is staining of heart tissue (HE, original magnification, 200 ×, Scale bar = 50 µm) (n = 3). **E** Levels of TNF-α and IL-6 in serum detected by ELISA kits (n = 10). **F**–**H** Levels of WBC, NEU and LYM in blood detected by blood analyzer (n = 10). ^#^*P* < 0.05, ^###^*P* < 0.005 *vs* Sham; ^*^P < 0.05, ^**^P < 0.01, ^***^P < 0.005 *vs* I/R

**Fig. 7:**
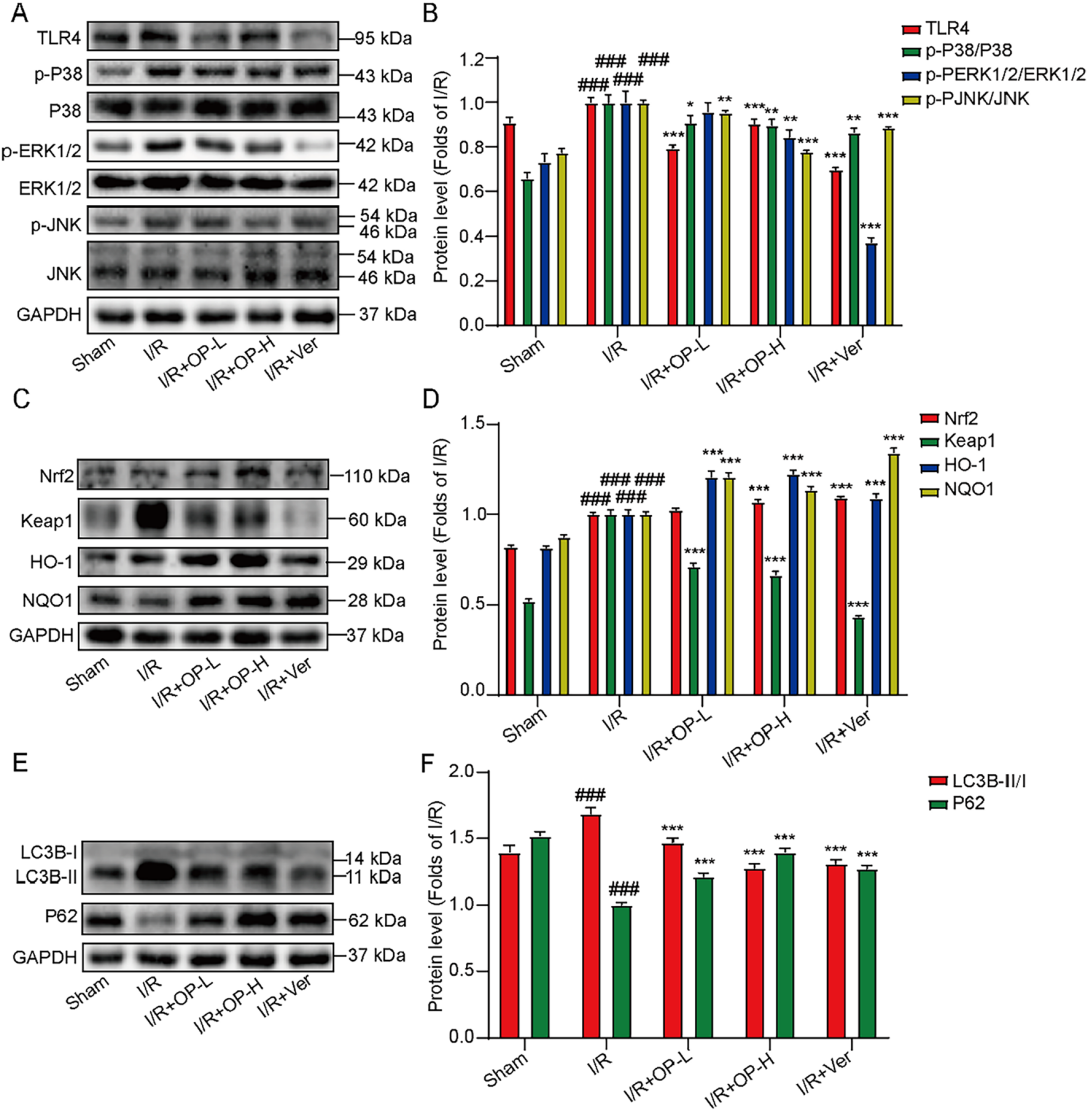
OP Regulates TLR4/MAPK/Nrf2 signaling pathway and autophagy in vivo **A**, **C**, **E** The protein expression of TLR4, p-P38. P38, p-ERK1/2, ERK1/2, p-JNK, JNK, LC3B, P62, Nrf2, Keap1, HO-1 and NQO in heart tissues were detected by western blotting (n = 4). **B**, **D**, **F** The statistics of protein levels of A, C, E. #*P* < 0.05, ###*P* < 0.005 vs Sham; *P < 0.05, **P < 0.01, ***P < 0.005 vs I/R

To further verify the therapeutic effect of OP on MIRI in vivo, cardiac ischemia–reperfusion surgery was performed after OP administration of rats for 7 days (Fig. 6A). The results showed that myocardial ischemia–reperfusion caused ST-segment elevation, myocardial infarction, myocardial cell swelling, nuclei marginalization, interstitial edema, muscle fiber rupture, and perinuclear inflammatory vacuoles. Nonetheless, OP significantly improved these injuries by lowing ST-segment, reducing infarct size, and suppressing damage to cardiac tissue and cardiomyocytes (Fig. 6B–D). Besides, myocardial ischemia–reperfusion increased the levels of TNF-α and IL-6 in the serum, and these levels were reduced by OP treatment (Fig. 6E). Furthermore, OP decreased the increased numbers of WBC, NEU, and LYM in the blood (Fig. 6F–H). In addition, cardiac tissue protein detection showed that ischemia/reperfusion increased the protein expression of TLR4, the ratio of LC3B-II/I, Keap1, and phosphorylation of p38, ERK1/2, and JNK as well as decreased P62 expression. OP further decreased TLR4, p-P38, p-ERK1/2, p-JNK and Keap1 levels and increased P62, Nrf2, HO-1 and NQO1 protein levels (Fig. 7). These data suggest that OP has a protective effect on MIRI, which may be carried out by regulating the TLR4/MAPK/Nrf2 pathway and autophagy.

## Discussion

Ischemia/reperfusion injury is unavoidable during the recovery of coronary blood flow and myocardial ischemia, and reduction of myocardial infarct size. With the wide application of various cardiac interventional therapy techniques, the cure rate of patients with cardiovascular diseases has greatly been improved, but the resulting ischemia/reperfusion injuries are also increasing [31]. Previous studies have found that OP has a protective effect on MIRI, but this study, for the first time, proves that OP can regulate oxidative stress and autophagy through the TLR4/MAPK pathway to exert cardioprotective effects [32].

Excessive production of oxidants during myocardial ischemia–reperfusion breaks the dynamic balance between oxidation and antioxidant defense systems, which is called oxidative stress. oxidative stress is often associated with elevated ROS or reactive nitrogen species (RNS) levels at the cellular and subcellular levels, wherein ROS acts more broadly [33]. ROS are highly active and short-lived substances produced in the body that are considered as important mediators of cell metabolism, proliferation, differentiation, immune system regulation and apoptosis [34]. Mitochondria are the main source of ROS production during MIRI [35]. Overloaded ROS induces the activation of the stress response pathway, causing the instability of mitochondria and sarcolemma, allowing non-specific small molecules to enter the membrane and resulting in mitochondrial matrix swelling and apoptosis. Furthermore, ROS overload can also directly lead to protein denaturation, cause DNA damage, interferes with DNA replication, and affects the correct synthesis of proteins [36]. In addition, ROS can even degrade components located in the intercellular space, increasing the permeability of the cell membrane, and consequently affecting the transport of substances, signal transmission, and other functions. All of these results in cardiomyocytes damage, and even death. Therefore, activating or enhancing the antioxidant defense system to prevent the damage caused by ROS is an important target of antioxidant drugs in the treatment of myocardial ischemia/reperfusion.

OP is an effective antioxidant [37]. In this study, tBHP was used to stimulate H9C2 cells to establish an in vitro oxidative stress model. The results showed that OP could reduce the ROS level and protect H9C2 cells from tBHP-induced injury. tBHP decreases the mitochondrial membrane potential, and this was reversed by OP treatment, reflecting that OP has a protective effect on the mitochondria which in leads to reduced ROS production (Fig. 1J). Additionally, OP activated the Nrf2 antioxidant system, promoted the expression and nuclear translocation of Nrf2, and then increased the expression of its downstream antioxidant enzymes HO-1 and NQO1, further reducing ROS generation and increasing ROS clearance (Fig. 2). Thus, OP could protect cardiomyocytes from oxidative stress damage through Nrf2 antioxidant pathways.

Autophagy plays an important role in MIRI [38]. Autophagy can degrade cytoplasmic proteins and organelles that have no important functions in cells, to provide nutrients for the key metabolic processes of cell survival [39]. From this perspective, autophagy has obvious cardioprotective effects in cardiomyocytes. However, in the stage of ischemia–reperfusion, the promoter of autophagy is mainly ROS. Excessively increased autophagy and overactivated autophagy-lysosomal protein degradation system degrades important intracellular proteins and organelles, causing irreversible damage to cells and even leading to cell death [40]. In this study, we found that OP has an inhibitory effect on tBHP-induced autophagy, and the autophagy inhibitor CQ also showed a protective effect on tBHP-stimulated H9C2 cells. CQ is a late autophagy inhibitor, which inhibits the fusion of autophagosomes and lysosomes into autolysosomes. The results showed that compared with the model group, the CQ group further increased the ratio of LC3BII/I and increased the P62 levels by blocking autophagic degradation, while OP increased the level of P62 but decreased LC3B levels, indicating that OP may inhibit the formation of autophagosomes at the early stage for inhibiting autophagy (Fig. 3). Taken together, OP also exerts a protective effect by inhibiting autophagy.

The MAPK signaling pathway constitutes the serine/threonine kinase family, which activates a wide range of extracellular stimuli and can generate different intracellular responses through transcriptional and non-transcriptional regulation [41]. The activation of MAPK signaling pathway is involved in the myocardial injury process in ischemia/reperfusion mice. Zeng et al. [42] found that notoginseng saponin R1 prevented MIRI in mice by inhibiting TAK1-JNK/p38 signaling. Jin et al. [43] further reported that dual-specificity protein phosphatase 1 (DUSP1) can protect mitochondrial homeostasis in mice with myocardial infarction, and its mechanism is associated with the inactivation of JNK signaling pathway. Pei et al. [44] reported that C-reactive protein can increase the infarct size of ischemia/reperfusion myocardial tissue through the continuous activation of the ERK1/2 signaling pathway. Therefore, inhibiting the activity of MAPK signaling pathways (P38, ERK1/2, and JNK) has a positive recovery effect on MIRI. In the study, OP exhibited an inhibitory effect on the phosphorylation of P38, ERK1/2, and JNK induced by tBHP (Fig. 4F, G).

TLRs are an evolutionarily conserved family of receptors that play a key role in activating the innate immune system in response to microbial invasion [45]. After being stimulated by corresponding ligands, intracellular adapter proteins are recruited to TLRs to form signaling complexes and subsequently mediate downstream signal transduction. TLR4 is one of the most well-studied members of the TLR family. In addition to exogenous lipopolysaccharide (LPS), various endogenous molecules released from damaged or ischemic tissues can also activate TLR4 signaling [46]. Thus, TLR4 has been identified as a mediator of inflammation and organ damage in several sterile tissue injury models, including ischemia/reperfusion injury [47].

TLR4 is the main mediator of myocardial injury and inflammation after ischemia/reperfusion [48]. Chong et al. found that TLR4 mutant mice exhibited significantly smaller infarcts, reduced activation level of JNK and significantly lower transcriptional levels of pro-inflammatory cytokines after ischemia/reperfusion [49]. Further, Lu et al. reported that astragaloside IV alleviated MIRI in rats by down-regulating the TLR4/NF-κB signaling pathway and inhibiting apoptosis [50]. Therefore, TLR4 has the potential to be a key target in the treatment of MIRI. The results of molecular docking, CETSA, and Biacore all indicated that there was a strong binding between OP and TLR4 (Fig. 4A–D). Moreover, OP could inhibit TLR4 expression. Subsequently, TLR4 inhibitor TAK242 could reduce tBHP-induced damage in H9C2 cells. Besides, TAK242 not only inhibits the TLR4/MAPK signaling pathway but also activates Nrf2 signaling pathway and inhibits autophagy, verifying that TLR4 plays a key role in regulating oxidative stress and autophagy in MIRI. Overall, all the abovementioned findings indicate that OP, as a TLR4 inhibitor, may protect against MIRI by targeting TLR4.

Tumor Necrosis Factor α (TNF-α) is an autocrine factor that participates in the formation and development of MIRI, and its expression level is increased during ischemia–reperfusion, which promotes ROS production and induces the sharp transcription of genes related to oxidative stress. Besides, TNF-α activates neutrophils and promotes adhesion and interaction between leukocytes and endothelial cells, increasing the infiltration of granulocytes into the ischemia–reperfusion area, resulting in myocardial damage [51]. Interleukin-6 (IL-6) is an important factor in the inflammatory storm, and is mainly produced by activated neutrophils in the pathological process. IL-6 can induce neutrophils to flow into the ischemic myocardial tissue and stimulate the expression of CDllb/ CDl8 on the surface of neutrophils and intercellular adhesion molecule 1 on cardiomyocytes in order to mediate the combination of the two. It plays a very important role in the process of acute myocardial infarction, and there is a positive correlation between the area of myocardial necrosis and IL-6 levels in the plasma [52]. Thus, the simultaneous inhibition of IL‐6, TNF‐α, WBC, NEU and LYM observed in OP-treated rats could also have mediated the anti‐MIRI effect of OP. Moreover, in vitro, OP exhibited a good protective effect in the rat model of myocardial ischemia/reperfusion by including reducing ST-segment elevation, reducing heart infarct size, and reducing the degree of myocardial damage (Fig. 6). OP treatment induced a significant reduction in the expression of TLR4, p-P38, p-ERK1/2, p-JNK, and Keap1 and the ratio of LC3B-II/I, and increased the levels of P62, Nrf2, HO-1 and NQO1 in heart tissues, which was consistent with the results of in vitro studies.

## Conclusion

**Fig. 8:**
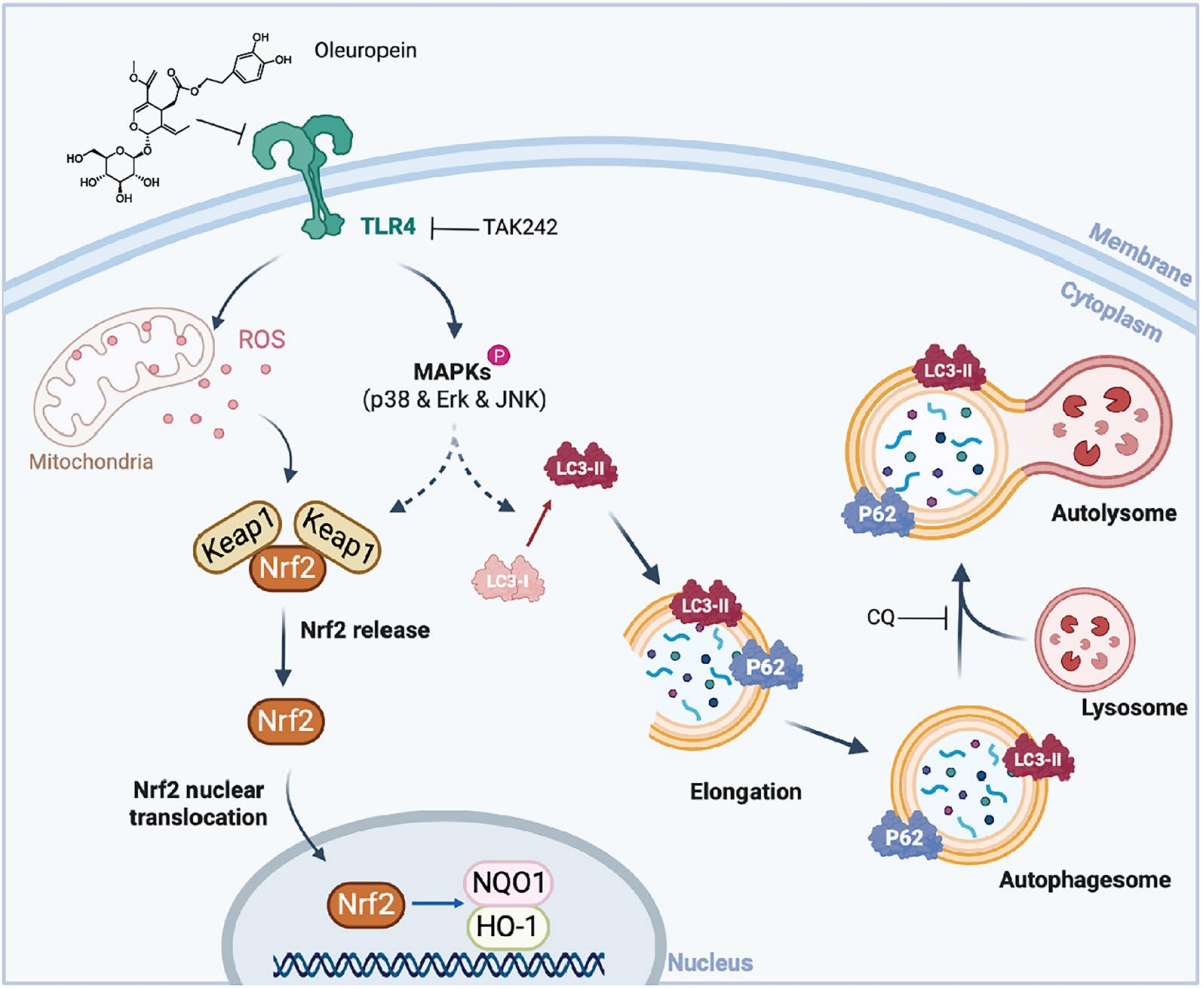
The molecular mechanism for OP regulating myocardial ischemia reperfusion

Taken together, our findings suggest that OP plays a protective role against MIRI by regulating oxidative stress and autophagy through the TLR4/MAPK signaling pathway (Fig. 8). Thus, OP may represent a new add‐on therapy for MIRI.

## Data Availability

All data generated or analysed during this study are included in this published article.

## Abbreviations

IHD: Ischemic heart disease
OP: Oleuropein
MIRI: Myocardial ischemia–reperfusion injury
TLR4: Toll-like receptor 4
MAPK: Mitogen-activated protein kinase
tBHP: Tert-Butyl hydroperoxide
ROS: Reactive oxygen species
MMP: Mitochondrial membrane potential
Nrf2: Nuclear factor erythroid 2-related factor 2
Keap1: Kelch-like ECH-associated protein 1
LC3B: Microtubule-associated proteins light chain 3B
ERK1/2: Extracellular signal‐regulated kinase1/2
JNK: C‐Jun N‐terminal kinases
GAPDH: Glyceraldehyde-3-phosphate dehydrogenase
TNF-α: Tumor necrosis factor‐ɑ
IL-6: Interleukin 6
WBC: White blood cell
NEU: Neutrophils
LYM: Lymphocytes
